# Generalized Pulse Width Modulation Switch Model for Converters Based on the Multistate Switching Cell in Discontinuous Conduction Mode

**DOI:** 10.3390/s24103084

**Published:** 2024-05-13

**Authors:** Fernando Lessa Tofoli

**Affiliations:** Department of Electrical Engineering, Federal University of São João del-Rei, São João del-Rei 36307-352, Brazil; fernandolessa@ufsj.edu.br

**Keywords:** dc–dc converters, discontinuous conduction mode, multistate switching cell, PWM switch model, small-signal modeling

## Abstract

This work introduces a generalized version of the pulse width modulation (PWM) switch model applied in the small-signal modeling of converters based on the multistate switching cell (MSSC) operating in discontinuous conduction mode (DCM). It consists of extending the concept formerly introduced by Vorperian for the representation of multiphase converters in DCM, yielding a circuit-based approach that does not rely on matrix manipulations unlike state-space averaging (SSA). The derived dc and ac models are valid for any number of switching states and any operating region defined in terms of the duty cycle, thus allowing for determining the voltage gain and distinct transfer functions. A thorough discussion of results is presented to demonstrate the applicability of the derived models to represent distinct configurations of the MSSC.

## 1. Introduction

Power electronic converters are complex nonlinear time-variant systems. Small-signal modeling approaches aim at linearizing them around an equilibrium point so that they can be treated as linear time-invariant systems, which can benefit from the use of linear controllers. In this context, several small-signal modeling techniques are properly discussed and compared in [1]. Although this topic has been extensively explored in the literature, it has been the focus of recent research. For instance, the authors in [2] derive the small-signal model of a multiphase buck converter based on current-mode constant on-time control (CMCOT) applied in voltage regulators in terms of a block diagram representation. However, this model does not seem to be an intuitive solution for deriving transfer functions. In turn, the work in [3] investigates the operation of a single-inductor dual-output (SIDO) buck converter operating in pseudocontinuous-conduction mode (PCCM) to achieve minimized cross-regulation. The four-switch buck–boost (FSBB) converter is represented in [4] by a nonlinear average model in which the average inductor current is not one of the state variables. Thus, it is possible to incorporate the influence of phase-shift variations into the model. The representation addressed in [5] relies on determining the state-space equation for the large-signal model either through circuit averaging or state-space averaging (SSA).

Overall, SSA is possibly the most popular approach for the small-signal modeling of power converters. It can be virtually applied to any topology operating in continuous conduction mode (CCM) or discontinuous conduction mode (DCM) [6,7]. However, SSA requires manipulating matrices for deriving transfer functions of interest, resulting in somewhat cumbersome equations that can be challenging to represent in a literal form even using powerful software tools. This is especially true in the case of high-order converters as demonstrated in [8,9].

In turn, the pulse width modulation (PWM) switch model is a far more intuitive solution. Similarly to SSA, it involves neglecting the high-frequency ripple of the current and voltage waveforms while applying small-signal perturbations to the average quantities. One can then represent the canonical cell or two-state switching cell (2SSC) in terms of an averaged model that can be used to analyze converters operating in CCM [10] or DCM [11]. One major advantage of this technique is that it relies solely on Ohm’s and Kirchhoff’s laws to perform the small-signal analysis as stated in [12].

The PWM switch model was first represented in the form of a single-pole, double-throw switch (SPDT) in [13]. It was also applied to the small-signal analysis of the classical non-isolated dc–dc converters in CCM [10] and DCM [11] in terms of two distinct averaged models. Later, it was extended to the modeling of power converters with many active switches in [14]. Alternate forms of the PWM switch in CCM and DCM are also described in [15,16], respectively.

However, an inherent limitation is that it can only represent hard-switched and quasi-resonant converters. In this sense, some improved versions of the PWM switch model have been proposed in the literature. For instance, it is possible to analyze quasi-resonant topologies by employing the modified circuit proposed in [17]. A more complete representation that takes into account the inductor in the modeling is also presented in [18], whereas a similar approach that allows for assessing the nonlinear and switching characteristics of semiconductors is presented in [19]. In turn, a general behavioral large-signal model capable of representing the operation in CCM and DMC based on a unified circuit is introduced in [20].

Other works in the literature have employed this concept for distinct purposes. For instance, sampled-data modeling is combined with pole-zero representation to derive an accurate current-mode control model in [21]. A similar three-terminal switch model for current mode control that preserves the current feedback information is presented in [22]. The current sharing control of paralleled converters based on the PWM switch model is proposed in [23]. In turn, one can design closed-loop control systems for the ac–dc Ćuk and single-ended primary inductance converter (SEPIC) rectifiers in CCM as in [24]. More advanced applications include the representation of modular dc–dc converters [25], the small-signal modeling of tapped inductor-based converters operating with hysteresis current control [26], and control of a single-phase active power filter [27].

The small-signal and transient analysis of more complex arrangements using the PWM switch is a possibility as demonstrated in [28], which focuses on a zero-voltage switching (ZVS) isolated dc–dc full-bridge converter. In addition, the influence of the transformer leakage inductance on the hard-switching version of the aforementioned topology is investigated in [29]. The synchronous buck converter is modeled in [30], whereas the PWM switch model is applied to the control of a single-phase boost inverter in [31]. More recently, a non-isolated high step-up boost converter based on the three-state switching cell (3SSC) was represented in terms of an equivalent boost converter for small-signal modeling purposes utilizing the PWM switch model in [32]. Later, the authors in [33] demonstrated that the transfer functions of the classical 3SSC-based converters in CCM are identical to the ones valid for their counterparts relying on the 2SSC.

A comprehensive investigation of the existing literature reveals a predominant emphasis on the PWM switch applied in the small-signal modeling of converters in CCM, with limited attention given to the operation in DCM. The authors in [34] propose a linear circuit model based on current-controlled current sources to perform the small-signal analysis of a dc–dc boost converter in DCM. Even though the model takes into account parasitics associated with the filter elements and semiconductors, this solution appears to be the simple application of the PWM switch model. Moreover, extending it to multiphase converters can be a somewhat complex task.

The work in [35] introduces a general method for developing state-space average-value models of PWM dc–dc converters operating in both CCM and DCM. Despite the authors’ claim that this method simplifies the consideration of circuit element parasitics, it still involves deriving state-space equations, similar to conventional SSA. The work reported in [36] utilizes the equivalent area method to establish an accurate and functional state-space averaged model applicable to both operating modes. However, this approach also requires matrix manipulations and complex calculations. The authors in [37] explore a circuit-based approach for analyzing dc–dc converters in DCM when supplying constant power loads. Nevertheless, deriving linearized models that include parasitics may result in somewhat cumbersome transfer functions.

Conversely, applying the PWM switch model to a dc–dc buck–boost converter as in [38] shows that it is very easy to incorporate parasitics such as the series resistance of the active switch and the forward voltage drop of the diode into the modeling. In this sense, a simple current mode control approach based on the PWM switch model is addressed in [39], thus demonstrating the great potential of this tool in performing the small-signal analysis in CCM and DCM.

After thorough analysis of the literature, it can be concluded that there is a lack of comprehensive representation of converters employing the multistate switching cell (MSSC), which is an extension of the 3SSC designed to derive multiphase converters intended for high-power, high-current applications. Considering the aforementioned gap, this study serves as a continuation of the research presented in [40], while presenting the following contributions to the state of the art of small-signal analysis:

- Introduction of a generalized form of the PWM switch that can be applied to the analysis of multiphase converters operating in DCM;
- Development of dc and ac models aiming to characterize converters employing the MSSC in DCM;
- Demonstrating the applicability of the unified models to the representation of MSSC-based converters while considering any number of switching states and any operating region defined in terms of the rated duty cycle;
- Validating the proposed models through a thorough analysis performed in both time and frequency domains.

The organization of this work is as follows: Section 2 presents a brief description of switching cells. Section 3 describes the proposed method, which in turn is applied to an MSSC-based buck converter in Section 4. Section 5 presents a comprehensive discussion of results obtained from the modeling. Section 6 concludes the study while pointing out possible future investigations on the subject.

## 2. Brief Theoretical Background

As previously mentioned, the PWM switch can be understood as an SPDT switch that constitutes the classical non-isolated dc–dc converters, that is, buck, boost, buck–boost, Ćuk, SEPIC, and Zeta. It consists of a two-port arrangement composed of three terminals: active (*a*), passive (*p*), and common (*c*). From this premise, observing the behavior of typical current and voltage waveforms allows for deriving an averaged circuit model as detailed in [10,11].

The 3SSC was proposed in [41] as a switching cell capable of generating ac–dc, dc–dc, and dc–ac converter topologies for high-power applications that involve high current levels. This representation relies on the interleaving principle, but an autotransformer with a unity turns ratio can provide improved current sharing without the need for special control schemes, unlike conventional two-phase interleaved topologies. The four-state switching cell (4SSC) is an extension of the 3SSC, which relies on a three-phase autotransformer and requires gating signals shifted by 120° for the active switches [42].

**Figure 1:**
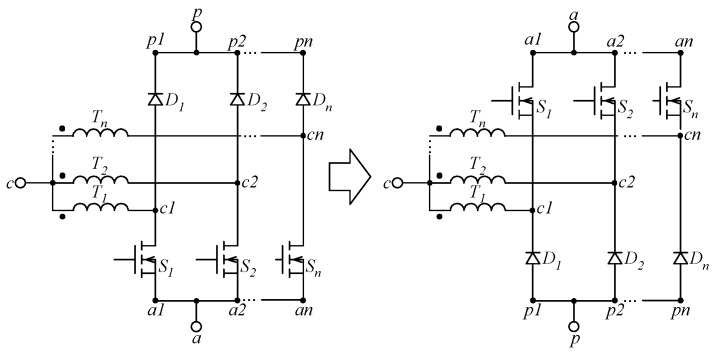
MSSC type B and its respective bilateral inversion.

In turn, the MSSC consists of generalizing the 3SSC and the 4SSC. Figure 1 shows that it relies on a multiphase autotransformer with a unity turns ratio connected to *r* = *M −* 1 legs or branches composed of active switches and diodes (or active switches only in bidirectional topologies), where *r* is the number of phases of the autotransformer and *M* is the number of switching states. The drive signals of the active switches are also shifted by 360°/*r*. Thus, one can achieve natural current sharing as long as the windings have the same impedance. The operating frequency of the filter elements becomes *r* times the switching frequency, consequently resulting in a higher power density. Analogously, the MSSC consists of *r* 2SSCs as displayed in Figure 1, while it can be used in the conception of novel converter topologies based on a general representation of the PWM switch.

The configuration to the left of Figure 1 shows that the source terminals of the metal-oxide-semiconductor field-effect transistors (MOSFETs) corresponding to switches *S*_1_…*S_n_* are connected to each other. Thus, there is no need for isolated gate drivers and/or dead time in this case. In turn, one can obtain the bilateral inversion of the MSSC by changing the position of diodes and switches, resulting in the configuration on the right of Figure 1. In either case, the very same properties and operating principles are maintained in the conception of converter topologies. The PWM switch model can also be promptly applied in this case to represent the resulting topologies. Thus, it is only necessary to replace the 2SSC with the MSSC to obtain other topologies that are adequate for high-current applications.

## 3. Unified Small-Signal Model of MSSC-Based Converters in DCM

### 3.1. DC Model

Deriving the PWM switch model as in [10,11] requires observing the waveforms associated with the terminals that constitute the canonical cell, which have the same behavior in all basic converters. This very same premise can be adopted in the analysis of MSSC-based topologies. However, such waveforms do change depending on the operating regions of the MSSC defined by the rated duty ratio as demonstrated in [41,42] for 3SSC- and 4SSC-based topologies, respectively.

As previously mentioned, the MSSC shown in Figure 1 relies on a multiphase autotransformer connected to *r* = *M −* 1 branches composed of active switches and diodes. The total number of operating regions *r* is a function of the number of switching states *M* according to (1), whereas the operation in a given region depends on the value assumed by *D* according to (2).

(1) r=M−1,

(2) $$\frac{R_{n}-1}{M-1}\leq D^{*}\leq \frac{R_{n}}{M-1},\;1\leq R_{n}\leq M-1,\;M\geq 2,\;M,R_{n}\in {\mathbb{N}}^{*}.$$

where D^*^ corresponds to a generic representation of the duty cycle of the active switch(es) in an MSSC-based converter and *R_n_* denotes a given operating region of the MSSC for *n* = 1, 2, …, *M* − 1.

**Figure 2:**
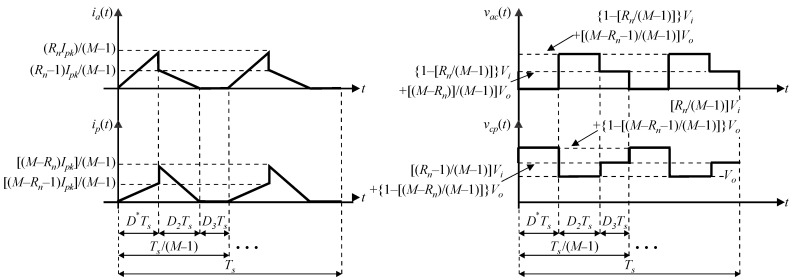
Waveforms of the instantaneous currents and voltages associated with the PWM switch considering the MSSC operating in DCM for a generic region *R_n_*.

Following the same reasoning as in [11], it is necessary to observe the behavior of the waveforms associated with terminals *a*, *c*, and *p* in Figure 1 while taking into account the common–common configuration corresponding to the dc–dc buck–boost converter. In particular, the instantaneous currents through terminals *a* and *p* corresponding to *i_a_*(*t*) and *i_p_*(*t*), respectively are of interest to the analysis, as well as the instantaneous voltages across terminals and *a*−*c* and *c*−*p*, represented by *v_ac_*(*t*) and *v_cp_*(*t*), respectively. It is noteworthy that Figure 2 corresponds to generic waveforms that represent the operation of the MSSC in DCM considering any operating region.

One can define the instantaneous waveforms shown in Figure 2 in terms of (3)–(6), which become valid for any region *R_n_* and any number of switching states of the MSSC.

(3) $$i_{a}\left(t\right)=\left\{\begin{array}{l} \left(\frac{R_{n}I_{pk}}{M-1}\right)\left(\frac{t}{D^{*}T_{s}}\right),\left[0,D^{*}T_{s}\right]\\ \left[\frac{\left(R_{n}-1\right)I_{pk}}{M-1}\right]\left(-\frac{t}{D_{2}T_{s}}+1\right),\left[0,D_{2}T_{s}\right]\\ 0,\left[0,D_{3}T_{s}\right] \end{array}\right.,$$

(4) $$i_{p}\left(t\right)=\left\{\begin{array}{l} \left[\frac{\left(M-R_{n}-1\right)I_{pk}}{M-1}\right]\frac{t}{D^{*}T_{s}},\left[0,D^{*}T_{s}\right]\\ \left[\frac{\left(M-R_{n}\right)I_{pk}}{M-1}\right]\left(-\frac{t}{D_{2}T_{s}}+1\right),\left[0,D_{2}T_{s}\right]\\ 0,\left[0,D_{3}T_{s}\right] \end{array}\right.,$$

(5) $$v_{ac}\left(t\right)=\left\{\begin{array}{l} \left(1-\frac{R_{n}}{M-1}\right)V_{i}+\left(\frac{M-R_{n}-1}{M-1}\right)V_{o},\left[0,D^{*}T_{s}\right]\\ \left(1-\frac{R_{n}-1}{M-1}\right)V_{i}+\left(\frac{M-R_{n}}{M-1}\right)V_{o}+,\left[0,D_{2}T_{s}\right]\\ V_{i},\left[0,D_{3}T_{s}\right] \end{array}\right.,$$

(6) $$v_{cp}\left(t\right)=\left\{\begin{array}{l} \left(\frac{R_{n}}{M-1}\right)V_{i}+\left(1-\frac{M-R_{n}-1}{M-1}\right)V_{o},\left[0,D^{*}T_{s}\right]\\ \left(\frac{R_{n}-1}{M-1}\right)V_{i}+\left(1-\frac{M-R_{n}}{M-1}\right)V_{o},\left[0,D_{2}T_{s}\right]\\ V_{o},\left[0,D_{3}T_{s}\right] \end{array}\right.,$$

where *I_pk_* is the peak inductor current; *D*_2_ and *D*_3_ are time intervals associated with the second and third operating stages in DCM, respectively; *T_s_* is the switching period; *V_i_* is the average input voltage; and *V_o_* is the average output voltage.

The average values of *i_a_*(*t*) and *i_p_*(*t*) corresponding to *I_a_* and *I_p_* can be calculated from (7) and (8), respectively.

(7) $$I_{a}=\frac{M-1}{T_{s}}\int_{0}^{T_{s}}i_{a}\left(t\right)dt=\frac{\left[R_{n}D^{*}+\left(R_{n}-1\right)D_{2}\right]I_{pk}}{2},$$

(8) $$I_{p}=\frac{M-1}{T_{s}}\int_{0}^{T_{s}}i_{p}\left(t\right)dt=\frac{\left[\left(M-R_{n}-1\right)D^{*}+\left(M-R_{n}\right)D_{2}\right]I_{pk}}{2}.$$

One can determine the average value of *v_ac_*(*t*) corresponding to *V_ac_* from (9).

(9) $$V_{ac}=\frac{M-1}{T_{s}}\int_{0}^{T_{s}}v_{ac}\left(t\right)dt=\left[\left(M-R_{n}-1\right)D^{*}+\left(M-R_{n}\right)D_{2}\right]\left(V_{i}+V_{o}\right)+\left(M-1\right)D_{3}V_{i}.$$

**Figure 3:**
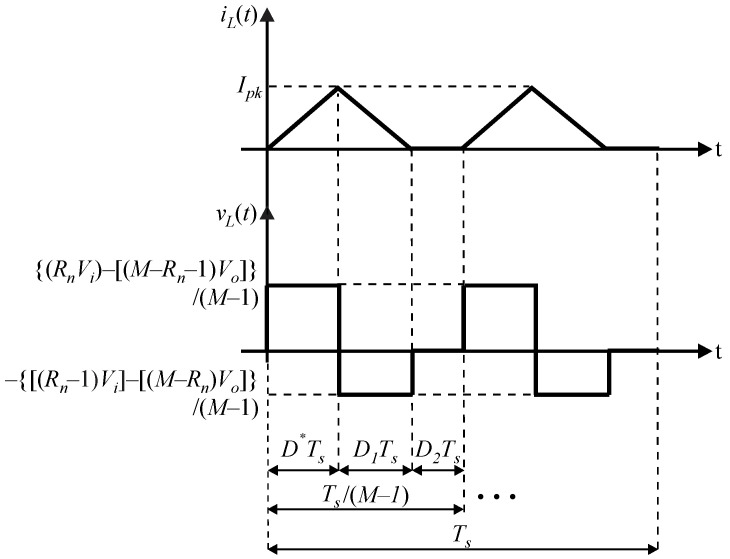
Waveforms of the filter inductor considering the MSSC operating in DCM for a generic region *R_n_*.

From the waveforms of the instantaneous current *i_L_*(*t*) and instantaneous voltage *v_L_*(*t*) of the filter inductor *L* in Figure 3, which are represented in terms of (10) and (11), respectively, one can write (12) employing the volt-second balance principle.

(10) $$i_{L}\left(t\right)=\left\{\begin{array}{l} \frac{I_{pk}t}{D^{*}T_{s}},\left[0,D^{*}T_{s}\right]\\ I_{pk}\left(-\frac{t}{D_{2}T_{s}}+1\right),\left[0,D_{2}T_{s}\right]\\ 0,\left[0,D_{3}T_{s}\right] \end{array}\right.,$$

(11) $$v_{L}\left(t\right)=\left\{\begin{array}{l} \frac{R_{n}V_{i}-\left(M-R_{n}-1\right)V_{o}}{M-1},\left[0,D^{*}T_{s}\right]\\ -\frac{\left(R_{n}-1\right)V_{i}-\left(M-R_{n}\right)V_{o}}{M-1},\left[0,D_{2}T_{s}\right]\\ 0,\left[0,D_{3}T_{s}\right] \end{array}\right.,$$

(12) $$\left[V_{i}-\left(\frac{V_{i}+V_{o}}{2}\right)\right]D^{*}\frac{T_{s}}{2}=V_{o}D_{2}\frac{T_{s}}{2}.$$

The equality given by (13) is also valid.

(13) $$D^{*}+D_{2}+D_{3}=\frac{1}{M-1}.$$

By isolating *D_2_* in (12) and *D_3_* in (13), as well as substituting both parameters in (9), one can obtain (14).

(14) $$V_{ac}=V_{i}.$$

From (14) and the equation that defines *v_L_*(*t*) in the first stage, one can write (15).

(15) $$V_{ac}=\left(\frac{M-R_{n}-1}{R_{n}}\right)V_{o}+\frac{\left(M-1\right)I_{pk}L}{R_{n}D^{*}T_{s}}.$$

The average value of *v_cp_*(*t*) corresponding to *V_cp_* can be calculated from (16).

(16) $$V_{cp}=\frac{M-1}{T_{s}}\int_{0}^{T_{s}}v_{cp}\left(t\right)dt=\left[R_{n}D^{*}+\left(R_{n}-1\right)D_{2}\right]\left(V_{i}+V_{o}\right)+\left(M-1\right)D_{3}V_{i}.$$

By isolating *D_2_* in (12) and *D_3_* in (13), as well as substituting both parameters in (16), it is possible to write (17).

(17) $$V_{cp}=V_{o}.$$

From (17) and the equation that defines *v_L_*(*t*) in the second stage, one can obtain (18).

(18) $$V_{cp}=\left(\frac{R_{n}-1}{M-R_{n}}\right)V_{ac}+\frac{\left(M-1\right)I_{pk}L}{\left(M-R_{n}\right)D_{2}T_{s}}.$$

Isolating *I_pk_* in (7) and (8) gives (19) and (20), respectively.

(19) $$I_{pk}=\frac{2I_{a}}{R_{n}D^{*}+\left(R_{n}-1\right)D_{2}},$$

(20) $$I_{pk}=\frac{2I_{p}}{\left(M-R_{n}-1\right)D^{*}+\left(M-R_{n}\right)D_{2}}.$$

Equaling (19) to (20) yields (21).

(21) $$I_{a}=\frac{\left[R_{n}D^{*}+\left(R_{n}-1\right)D_{2}\right]I_{p}}{\left(M-R_{n}-1\right)D^{*}+\left(M-R_{n}\right)D_{2}}.$$

One can represent D^*^ for a given region and a given number of switching states according to (22).

(22) $$D^{*}=D-\frac{R_{n}-1}{M-1}.$$

Substituting (22) in (21), the average current *I_a_* can be represented by (23).

(23) $$I_{a}=\frac{\left(M-1\right)\left[R_{n}D+\left(R_{n}-1\right)D_{2}\right]-R_{n}\left(R_{n}-1\right)}{\left(M-1\right)\left[\left(M-R_{n}-1\right)D+\left(M-R_{n}\right)D_{2}\right]-\left(M-R-1\right)\left(R_{n}-1\right)}I_{p}.$$

Isolating *I_pk_* in (15) and (18) while considering the equality in (17) results in (24) and (25), respectively.

(24) $$I_{pk}=\frac{D^{*}T_{s}\left[R_{n}V_{ac}-\left(M-R_{n}-1\right)V_{cp}\right]}{\left(M-1\right)L},$$

(25) $$I_{pk}=\frac{D_{2}T_{s}\left[-\left(R_{n}-1\right)V_{ac}+\left(M-R_{n}\right)V_{cp}\right]}{\left(M-1\right)L}.$$

Equaling (24) to (25), one can obtain (26). In turn, substituting (22) in (26) yields (27).

(26) $$V_{ac}=\frac{\left(M-R_{n}-1\right)D^{*}+\left(M-R_{n}\right)D_{2}}{R_{n}D^{*}+\left(R_{n}-1\right)D_{2}}V_{cp},$$

(27) $$V_{ac}=\frac{\left(M-1\right)\left(M-R_{n}-1\right)D+\left(M-1\right)\left(M-R_{n}\right)D_{2}-\left(R_{n}-1\right)\left(M-R_{n}-1\right)}{\left(M-1\right)\left[R_{n}D+\left(R_{n}-1\right)D_{2}\right]-R_{n}\left(R_{n}-1\right)}V_{cp}.$$

Substituting (15), (19), and (28) in (26) gives (29).

(28) $$f_{s}=\frac{1}{T_{s}},$$

(29) $$D_{2}=\frac{2I_{a}Lf_{s}}{D^{*}V_{cp}},$$

where *f_s_* is the switching frequency.

Substituting (18), (20), and (28) in (26) gives (30).

(30) $$D_{2}=\frac{2I_{p}Lf_{s}}{D^{*}V_{ac}}.$$

Equaling (29) to (30) yields (31).

(31) $$V_{ac}=\frac{I_{p}V_{cp}}{I_{a}}.$$

Substituting (23) in (31), one can obtain (32).

(32) $$V_{cp}=\frac{R_{n}\left[\left(M-1\right)D-\left(R_{n}-1\right)\right]+\left(M-1\right)\left(R_{n}-1\right)D_{2}}{\left(M-1\right)\left(M-R_{n}-1\right)D+\left(M-1\right)\left(M-R_{n}\right)D_{2}-\left(R_{n}-1\right)\left(M-R_{n}-1\right)}V_{ac}.$$

One can define parameter *μ* as in (33).

(33) $$\mu =\frac{R_{n}\left[\left(M-1\right)D-\left(R_{n}-1\right)\right]+\left(M-1\right)\left(R_{n}-1\right)D_{2}}{\begin{array}{l} \left(M-1\right)\left(M-R_{n}-1\right)D+\left(M-1\right)\left(M-R_{n}\right)D_{2}\\ -\left(R_{n}-1\right)\left(M-R_{n}-1\right) \end{array}}.$$

Substituting (22) in (29), as well as the resulting equation in (33), one can write (34).

(34) $$\mu =\frac{R_{n}{\left[\left(M-1\right)D-\left(R_{n}-1\right)\right]}^{2}V_{cp}+2{\left(M-1\right)}^{2}\left(R_{n}-1\right)I_{a}Lf_{s}}{\left(M-R_{n}-1\right){\left[\left(M-1\right)D-\left(R_{n}-1\right)\right]}^{2}V_{cp}+2{\left(M-1\right)}^{2}\left(M-R_{n}\right)I_{a}Lf_{s}},$$

Substituting (22) in (30), as well as the resulting equation in (33), one can write (35).

(35) $$\mu =\frac{R_{n}{\left[\left(M-1\right)D-\left(R_{n}-1\right)\right]}^{2}V_{ac}+2{\left(M-1\right)}^{2}\left(R_{n}-1\right)I_{p}Lf_{s}}{\left(M-R_{n}-1\right){\left[\left(M-1\right)D-\left(R_{n}-1\right)\right]}^{2}V_{ac}+2{\left(M-1\right)}^{2}\left(M-R_{n}\right)I_{p}Lf_{s}}.$$

**Figure 4:**
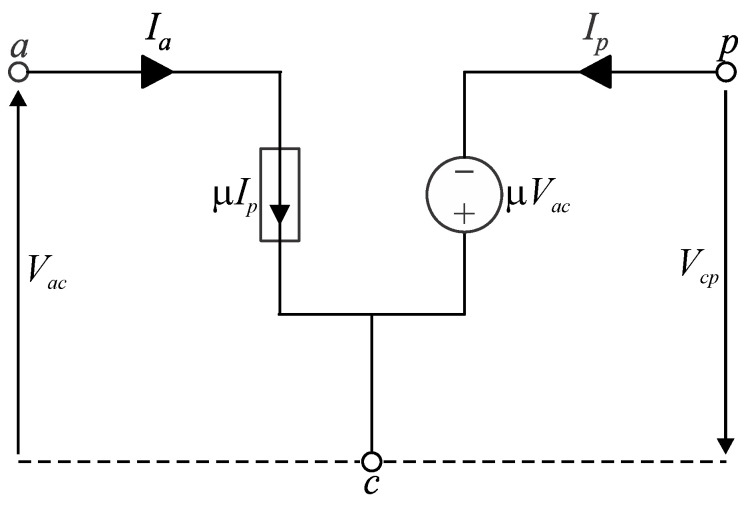
DC model of the PWM switch for representing MSSC-based converters operating in any region and DCM.

From (23), (32), and (33), one can write (36) and (37), which are the very same expressions that define the dc model of converters based on the 2SSC as presented in [11]. However, it is noteworthy that the corresponding circuit in Figure 4 is a generic representation valid for any values of *R_n_* and *M* as denoted by parameter *μ* in (33)–(35).

(36) $$I_{a}=\mu I_{p}.$$

(37) $$V_{cp}=\mu V_{ac}.$$

### 3.2. AC Model

Substituting (35) in (36) and perturbing all parameters allows for deriving the generalized ac model of the PWM switch for MSSC-based converters in DCM. After that, the resulting equation must be differentiated with respect to time while neglecting the terms related to the products of small-signal perturbations. For the sake of simplicity, a step-by-step procedure will not be presented here. Considering that the first-order derivatives of the variables with respect to time are denoted by “^” and after some algebra, one can obtain (38)–(41).

(38) $${\hat{i}}_{a}=k_{i}\hat{d}+g_{i}{\hat{v}}_{ac}+k_{c}{\hat{i}}_{p},$$

where

(39) $$k_{i}=\frac{2\left(M-1\right)V_{ac}\left[\left(M-1\right)D-\left(R-1\right)\right]\left[RI_{p}-\left(M-R_{n}-1\right)I_{a}\right]}{\left\{\left(M-R_{n}-1\right){\left[\left(M-1\right)D-\left(R_{n}-1\right)\right]}^{2}V_{ac}+2{\left(M-1\right)}^{2}\left(M-R_{n}\right)I_{p}Lf_{s}\right\}},$$

(40) $$g_{i}=\frac{{\left[\left(M-1\right)D-\left(R_{n}-1\right)\right]}^{2}\left[R_{n}I_{p}-\left(M-R_{n}-1\right)\right]}{\left\{\left(M-R_{n}-1\right){\left[\left(M-1\right)D-\left(R_{n}-1\right)\right]}^{2}V_{ac}+2{\left(M-1\right)}^{2}\left(M-R_{n}\right)I_{p}Lf_{s}\right\}},$$

(41) $$k_{c}=\frac{\left\{R_{n}{\left[\left(M-1\right)D-\left(R_{n}-1\right)\right]}^{2}V_{ac}-2{\left(M-1\right)}^{2}Lf_{s}\left[\left(M-R_{n}\right)I_{a}-2\left(R_{n}-1\right)I_{p}\right]\right\}}{\left\{\left(M-R_{n}-1\right){\left[\left(M-1\right)D-\left(R_{n}-1\right)\right]}^{2}V_{ac}+2{\left(M-1\right)}^{2}\left(M-R_{n}\right)I_{p}Lf_{s}\right\}}.$$

Analyzing (38) shows that the third term of the sum associated with ${\hat{i}}_{p}$ and represented by (41) does not exist in the former ac model derived in [11] for the classical non-isolated dc–dc converters in DCM; that is, *k_c_* = 0 in the 2SSC (*M* = 2, *R_n_* = 1). This is because the 2SSC comprises a single operating region where 0 ≤ *D* ≤ 1.

Similarly, substituting (35) in (37), perturbing all variables, manipulating the resulting equation, and differentiating it with respect to time while neglecting the terms related to the products of small-signal perturbations yields (42)–(45).

(42) $${\hat{i}}_{p}=g_{f}{\hat{v}}_{ac}+k_{o}\hat{d}-g_{o}{\hat{v}}_{cp},$$

where

(43) $$g_{f}=\frac{{\left[\left(M-1\right)D-\left(R_{n}-1\right)\right]}^{2}\left[2R_{n}V_{ac}-\left(M-R_{n}-1\right)V_{cp}\right]+2\left(R_{n}-1\right){\left(M-1\right)}^{2}I_{p}Lf_{s}}{2{\left(M-1\right)}^{2}Lf_{s}\left[\left(M-R_{n}\right)V_{cp}-\left(R_{n}-1\right)V_{ac}\right]},$$

(44) $$k_{o}=\frac{V_{ac}\left[\left(M-R_{n}-1\right)V_{cp}-R_{n}V_{ac}\right]\left[\left(M-1\right)D-\left(R_{n}-1\right)\right]}{\left(M-1\right)Lf_{s}\left[\left(R_{n}-1\right)V_{ac}-\left(M-R_{n}\right)V_{cp}\right]},$$

(45) $$g_{o}=\frac{\left(M-R_{n}-1\right){\left[\left(M-1\right)D-\left(R_{n}-1\right)\right]}^{2}V_{ac}+2\left(M-R_{n}\right){\left(M-1\right)}^{2}I_{p}Lf}{2{\left(M-1\right)}^{2}Lf_{s}\left[\left(M-R_{n}\right)V_{cp}-\left(R_{n}-1\right)V_{ac}\right]}.$$

**Figure 5:**
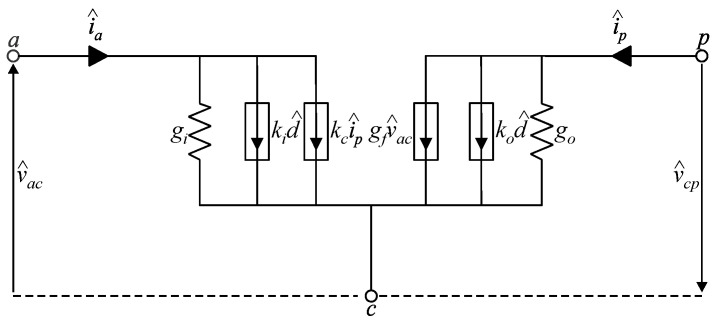
AC model of the PWM switch for representing MSSC-based converters operating in any region and DCM.

It is possible to represent the unified ac model of the MSSC in DCM in terms of the circuit shown in Figure 5. It is noteworthy that the model is not identical to the one derived in [11], even though it can be used for representing the 2SSC, 3SSC, 4SSC, or any other multistate configuration in DCM while also considering any value of the duty cycle and any operating region.

## 4. Small-Signal Modeling of a DC–DC Buck Converter Based on the MSSC in DCM

According to the methodology described in [7], applying SSA to the conventional dc–dc buck converter in DCM yields (46)–(52).

(46) $$\frac{v_{o}\left(s\right)}{d\left(s\right)}=G_{do}\frac{1+\frac{s}{\omega_{z}}}{1+\frac{s}{\omega_{p}}}$$

(47) $$G_{do}=\frac{2V_{o}}{D}\frac{1-G}{2-G},$$

(48) $$D=G\sqrt{\frac{K}{1-G}},$$

(49) $$G=\frac{V_{o}}{V_{i}},$$

(50) $$K=\frac{2Lf_{s}}{R_{o}},$$

(51) $$\omega_{p}=\frac{2-G}{1-G}\frac{1}{R_{o}C},$$

(52) $$\omega_{z}=\frac{1}{R_{SE}C},$$

where *G* is the voltage gain, *R_o_* is the load resistance, *C* is the output filter capacitance, *ω_p_* is the pole angular frequency, and *ω_z_* is the zero angular frequency.

The analysis of the control-to-output transfer function *v_o_*(*s*)/*d*(*s*) corresponding to (46) shows that it is a first-order system. This result is commonly presented as acceptable in most didactic books on power electronics. However, it was proven inaccurate in [11] while deriving (53)–(57).

(53) $$\frac{v_{o}\left(s\right)}{d\left(s\right)}=H_{d}\frac{1+\frac{s}{\omega_{z}}}{1+a_{1}s+a_{2}s^{2}}$$

(54) $$H_{d}=\frac{2V_{o}}{DR_{o}}\frac{r^{*}R_{o}}{r+R_{o}+R_{L}},$$

(55) $$r^{*}=\frac{1}{g_{i}+g_{o}+g_{f}},$$

(56) $$a_{1}=\frac{L}{r^{*}+R_{L}+R_{o}}+C\left[\left(R_{SE}+R_{o}\right)|\left(r^{*}+R_{L}\right)\right],$$

(57) $$a_{2}=LC\frac{R_{SE}+R_{o}}{r^{*}+R_{L}+R_{o}},$$

where *R_L_* is the series resistance of the filter inductor *L* and *R_SE_* is the equivalent series resistance (ESR) of the output filter capacitor *C*.

Substituting the PWM switch model in DCM in the buck converter reveals that the system has indeed two real poles. The first pole *f_p_*_1_ is the same as that obtained with SSA while the second pole *f_p_*_2_ occurs at a frequency *f_p_*_2_ ≥ *f_s_*/π. The impact of the second pole on the phase response can be substantial and readily confirmed within the frequency range below half the switching frequency. Additionally, given that the second pole aligns with a time constant shorter than half the switching period, its influence on the transient response diminishes within a single switching period or less. Consequently, the transient response observed is predominantly governed by the first pole. This incoherence was only eliminated after the introduction of the modified SSA technique in [43], which allows for deriving full-order models.

**Figure 6:**
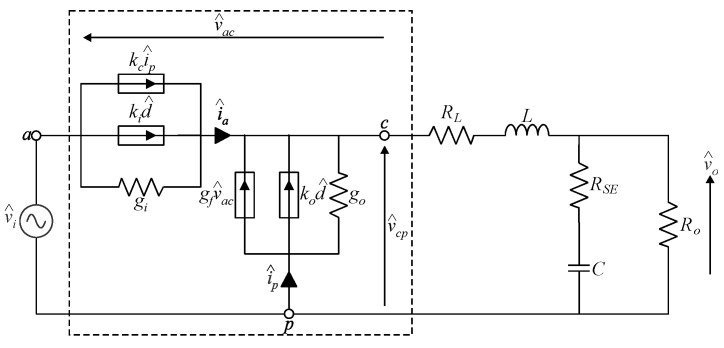
AC model of the PWM switch applied to the MSSC-based buck converter operating in DCM.

In turn, substituting the ac model shown in Figure 5 in the buck converter results in Figure 6. This representation differs from the one derived in [11] in two aspects: (i) it comprises an additional term $k_{c}{\hat{i}}_{p}$; and (ii) it consists of a generalized model valid for any values of *M* and *R_n_*. In other words, the ac model can be used to obtain the transfer functions of the buck converter shown in Figure 5 while considering any number of phases and branches associated with the MSSC in Figure 1, as well as any operating region.

Perturbing the duty cycle and observing its influence on the output voltage, as well as considering that the remaining variables are constant, one can obtain the control-to-output transfer function *v_o_*(*s*)/*d*(*s*) in (58). This is still a second-order system represented as a function of the model coefficients given by (38)–(41) and (43)–(45). Substituting *M* = 2 and *R_n_* = 1 in the aforementioned equations and rearranging the result yields (53) for the 2SSC-based buck converter, this being a particular case of (58). Other important results can also be obtained from Figure 5, including the input-to-output transfer function *v_o_*(*s*)/*v_i_*(*s*), the inductor-current-to-output transfer function *v_o_*(*s*)/*i_L_*(*s*), the control-to-inductor-current transfer function *i_L_*(*s*)/*d*(*s*), the input impedance *Z_i_*(*s*), and the output impedance *Z_o_*(*s*) in (58)–(63).

(58) $$\frac{v_{o}\left(s\right)}{d\left(s\right)}=\frac{R_{o}\left[\left(k_{c}+1\right)k_{o}+k_{i}\right]\left(CR_{SE}s+1\right)}{\begin{array}{l} \left\{\left[\left(k_{c}+1\right)\left(g_{f}+g_{o}\right)+g_{i}\right]\left(R_{o}+R_{SE}\right)\right\}LCs^{2}\\ +\left\{\left[\left(k_{c}+1\right)\left(g_{f}+g_{o}\right)+g_{i}\right]\left\{L+C\left[R_{o}\left(R_{L}+R_{SE}\right)+R_{L}R_{SE}\right]\right\}+C\left(R_{o}+R_{SE}\right)\right\}s\\ +\left\{\left[\left(k_{c}+1\right)\left(g_{f}+g_{o}\right)+g_{i}\right]\left(R_{o}+R_{L}\right)+1\right\} \end{array}},$$

(59) $$\frac{v_{o}\left(s\right)}{v_{i}\left(s\right)}=\frac{R_{o}\left[\left(k_{c}+1\right)g_{f}+g_{i}\right]\left(CR_{SE}s+1\right)}{\begin{array}{l} \left[\left(k_{c}+1\right)\left(g_{o}+g_{f}\right)+g_{i}\right]\left(R_{o}+R_{SE}\right)LCs^{2}\\ +\left\{\left[\left(k_{c}+1\right)\left(g_{o}+g_{f}\right)+g_{i}\right]\left\{L+C\left[R_{L}\left(R_{o}+R_{SE}\right)+R_{o}R_{SE}\right]\right\}+C\left(R_{o}+R_{SE}\right)\right\}s\\ +\left\{\left[\left(k_{c}+1\right)\left(g_{o}+g_{f}\right)+g_{i}\right]\left(R_{o}+R_{L}\right)+1\right\} \end{array}},$$

(60) $$\frac{v_{o}\left(s\right)}{i_{L}\left(s\right)}=\frac{R_{o}\left(R_{SE}Cs+1\right)}{\left(R_{o}+R_{SE}\right)Cs+1},$$

(61) $$\frac{i_{L}\left(s\right)}{d\left(s\right)}=\frac{\left(k_{c}+1\right)k_{o}+k_{i}}{Z_{eq}\left[\left(k_{c}+1\right)\left(g_{f}+g_{o}\right)g_{i}\right]+1},$$

(62) $$Z_{i}\left(s\right)=\frac{Z_{eq}\left[\left(k_{c}+1\right)\left(g_{o}+g_{f}\right)+g_{i}\right]+1}{g_{i}\left(Z_{eq}g_{o}+1\right)+g_{f}k_{c}},$$

(63) $$Z_{o}\left(s\right)=\frac{\left(R_{o}R_{SE}LC\right)s^{2}+\left[R_{o}\left(R_{L}R_{SE}C+L\right)\right]s+R_{o}R_{L}}{\left[\left(R_{o}+R_{SE}\right)LC\right]s^{2}+\left\{\left[\left(R_{L}+R_{SE}\right)R_{o}+R_{L}R_{SE}\right]C+L\right\}s+\left(R_{o}+R_{L}\right)},$$

where the equivalent impedance *Z_eq_* is given by (64).

(64) $$Z_{eq}=\left(R_{L}+sL\right)+\left[\frac{R_{o}\left(R_{SE}+\frac{1}{sC}\right)}{R_{o}+\left(R_{SE}+\frac{1}{sC}\right)}\right].$$

## 5. Results and Discussion

First, let us prove Vorperian’s point about the control-to-output transfer function of the conventional buck converter in DCM (*M* = 2, *R_n_* = 1, 0 ≤ *D* ≤ 1) by considering the following operating point: *V_i_* = 100 V, *V_o_* = 62.91 V, *D =* 0.4, *R_o_ =* 10 Ω, *f_s_* = 30 kHz, *L* = 25 µH, *R_L_* = 1 mΩ, *C =* 100 µF, and *R_SE_* = 10 mΩ. The filter inductance in this case is below the critical inductance; that is, the minimal value capable of ensuring the operation in CCM.

**Figure 7:**
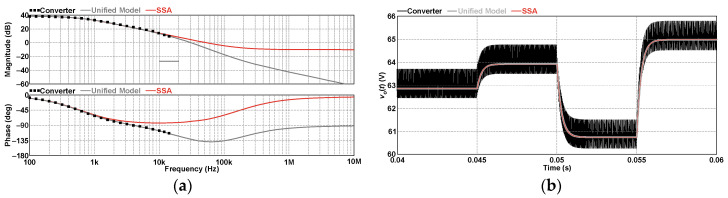
Control-to-output transfer function of the 2SSC-based buck converter in DCM: (**a**) frequency domain and (**b**) time domain.

Figure 7a compares the Bode plots of the small-signal models represented by (46) and (58). Although the small-signal models obtained from both SSA and the PWM switch method can represent the converter only up to half of the switching frequency, corresponding to 15 kHz in this case, the curves representing the transfer functions are plotted here within the range of 100 Hz to 10 MHz to highlight the differences in magnitude and phase. It is noteworthy that (46) has one pole *f_p_* = 588.19 Hz while (58) has two poles *f_p_*_1_ = 588.19 Hz and *f_p_*_2_ = 23.16 kHz. The difference between the plots is noticeable considering that the second pole of (58) causes a significant change in the phase response. In turn, analyzing the converter response obtained by simulation with ac sweep between 100 Hz and 10 kHz reveals that the magnitudes of the converter and the small-signal models nearly overlap within this range. In addition, the frequency response of the converter matches (58) better than (46). However, no significant differences are observed in the time responses of the models shown in Figure 7b when the duty cycle is perturbed by +0.01, –0.02, and +0.02 at 45 ms, 50 ms, and 55 ms, respectively. This is why the control-to-output transfer function was erroneously considered to be of first order.

**Figure 8:**
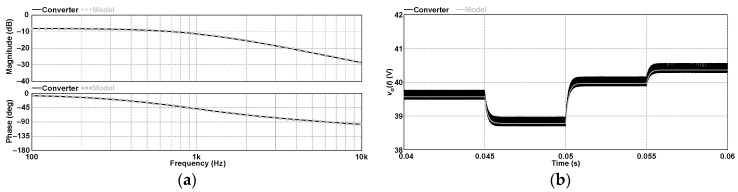
Input-to-output transfer function of the 3SSC-based buck converter in DCM and region *R*_1_: (**a**) frequency domain and (**b**) time domain.

To validate the proposed unified model, let us analyze the dc–dc buck converter based on the 3SSC described in [41] operating in DCM and region *R_1_* (*M* = 3, *R_n_* = 1, 0 ≤ *D* ≤ 1/2) while considering the following operating conditions: *V_i_* = 100 V, *V_o_* = 39.56 V, *D =* 0.3, *R_o_ =* 10 Ω, *f_s_* = 30 kHz, *R_L_* = 1 mΩ, *L* = 10 µH, *R_SE_* = 10 mΩ, and *C =* 100 µF. Substituting all the parameters in (59) allows for obtaining the numerical representation of the input-to-output transfer function, which is validated in both frequency and time domains in Figure 8 considering that the model follows the behavior of the switched converter accurately. It is also noteworthy that substituting *M* = 3 and *R_n_* = 1 or *M* = 3 and *R_n_* = 2 in the equations that provide the coefficients of the ac model represented in Figure 5 will lead to the very same results found in [40] for the 3SSC-based buck converter in DCM operating in the non-overlapping mode (NOM) or the overlapping mode (OM), respectively.

**Figure 9:**
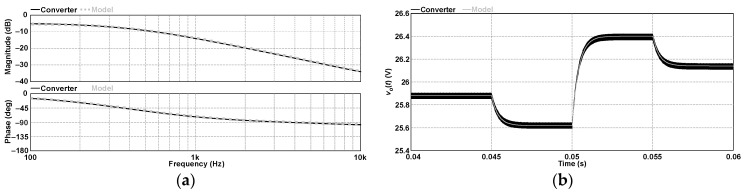
Input-to-output transfer function of the 4SSC-based buck converter in DCM and region *R*_2_: (**a**) frequency domain and (**b**) time domain.

Now, let us assess the dc–dc buck converter based on the 4SSC described in [42] operating in DCM and region *R_2_* (*M* = 4, *R_n_* = 2, 1/3 ≤ *D* ≤ 2/3) to demonstrate that the model is valid for other configurations of the MSSC while considering the following specifications: *V_i_* = 50 V, *V_o_* = 25.88 V, *D =* 0.45, *R_o_ =* 30 Ω, *f_s_* = 30 kHz, *L* = 10 µH, *R_L_* = 1 mΩ, *C =* 100 µF, and *R_SE_* = 10 mΩ. Figure 9a shows the behavior of the input-to-output transfer function corresponding to (59), evidencing that the curves are overlapped. In addition, the output voltage changes accordingly in Figure 9b when the rated input voltage is incremented by –0.5 V, +1 V, and +0.5 V at 45 ms, 50 ms, and 55 ms, respectively.

As another exercise for demonstrating the applicability of the proposed unified model, let us assess a dc–dc buck converter based on the five-state switching cell (5SSC), which remains unexplored in the literature thus far. Considering Figure 1 as a reference, one can state that the 5SSC consists of a four-phase autotransformer with a unity turns ratio, in turn, associated with four legs composed of one active switch and one diode each. The gating signals of the main switches are shifted by 360°/4 = 90° in this case, resulting in four distinct operating regions: *R*_1_ (0 ≤ *D* ≤ 1/4), *R*_2_ (1/4 ≤ *D* ≤ 2/4), *R*_3_ (2/4 ≤ *D* ≤ 3/4), and *R*_4_ (3/4 ≤ *D* ≤ 1).

First, it is necessary to define the following operating point in DCM (*M* = 5, *R_n_* = 3, 2/4 ≤ *D* ≤ 3/4): *V_i_* = 50 V, *V_o_* = 30.42 V, *D =* 0.6, *R_o_ =* 30 Ω, *f_s_* = 30 kHz, *L* = 10 µH, *R_L_* = 1 mΩ, *C =* 100 µF, and *R_SE_* = 10 mΩ. The objective here is to employ (60) and (61) to design the voltage and current control loops, respectively, considering the converter operating in average current mode control [44]. The loop responsible for controlling the inductor current must be fast, whereas the loop that regulates the output voltage must be slow, yielding a cascade control approach.

A simple tuning method for the controllers is the K factor [45], but a detailed design procedure will be omitted here for simplicity. Thus, the compensated current control loop was designed for a unity gain crossover frequency of *f_s_*/8 = 3.75 kHz and a phase margin of 77.5°. In turn, the compensated voltage control loop has a crossover frequency of 300 Hz and a phase margin of 90°.

**Figure 10:**
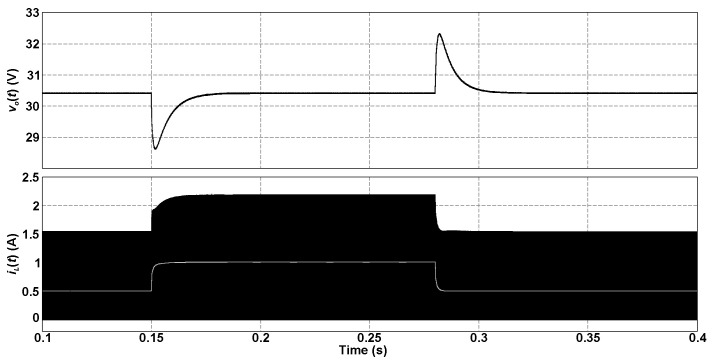
Behavior of the output voltage and inductor current of the 5SSC-based buck converter operating in average current mode control when the load power varies.

Figure 10 shows the behavior of the output voltage and inductor current waveforms when load steps from 50% to 100% of the rated power and vice versa occur at *t* = 0.15 s and *t* = 0.28 s, respectively. The inductor current becomes zero throughout the switching period, denoting the operation in DCM. The output voltage changes slowly as expected, even though it remains regulated after the disturbances as desired, thus demonstrating the accurate design of the control system.

## 6. Conclusions

This work has extended the PWM switch concept to the representation of non-isolated dc–dc converters employing the MSSC and operating in DCM. Considering a given number of switching states and a given operating region represented in terms of the value assumed by the rated duty ratio, it has been demonstrated that it is possible to calculate the parameters of the PWM switch model using generic equations that are valid for any condition.

The derived expressions corroborate the results previously reported by Vorperian for the 2SSC-based dc-dc buck converter. However, it is noteworthy that there is an additional term in the ac model associated with the current in the passive terminal for any *M* > 2, *M* ∈ ${\mathbb{N}}^{*}$.

From the unified models, one can easily calculate the voltage gain and transfer functions for any values of *M* and *R_n_*. Future work on the subject should include investigating other configurations of the 3SSC and 4SSC, as well as applying the methodology for deriving unified models of other topologies like non-isolated high step-up dc–dc converters operating in both CCM and DCM.

## Data Availability

Data are available upon request from the authors.
